# *Escherichia coli* killing by epidemiologically successful sublineages of *Shigella sonnei* is mediated by colicins

**DOI:** 10.1016/j.ebiom.2023.104822

**Published:** 2023-10-06

**Authors:** P. Malaka De Silva, Rebecca J. Bennett, Lauriane Kuhn, Patryk Ngondo, Lorine Debande, Elisabeth Njamkepo, Brian Ho, François-Xavier Weill, Benoît S. Marteyn, Claire Jenkins, Kate S. Baker

**Affiliations:** a Department of Clinical Infection, Microbiology, and Immunology, Institute for Infection, Veterinary, and Ecological Sciences (IVES), University of Liverpool, Liverpool, United Kingdom; b Plateforme protéomique Strasbourg Esplanade FR1589 du CNRS, Université de Strasbourg, Strasbourg, France; c Université de Strasbourg, CNRS, Architecture et Réactivité de l’ARN, UPR9002, F-67000, Strasbourg, France; d Institut Pasteur, Université Paris Cité, Unité des Bactéries pathogènes entériques, Centre National de Référence des *Escherichia coli*, *Shigella* et *Salmonella*, Paris, F-75015, France; e Institute of Structural and Molecular Biology, University College London and Birkbeck, London, UK; f Gastro and Food Safety (One Health) Division, UK Health Security Agency, Colindale, London, UK; g Department of Genetics, University of Cambridge, Downing Place, Cambridge, UK

**Keywords:** Shigella, Colicins, Interbacterial competition, T6SS

## Abstract

**Background:**

*Shigella* sp. are enteric pathogens which causes >125 million cases of shigellosis annually. *S. sonnei* accounts for about a quarter of those cases and is increasingly prevalent in industrialising nations. Being an enteric pathogen, *S. sonnei* benefits from outcompeting gut commensals such as *Escherichia coli* to establish itself and cause disease. There are numerous mechanisms that bacterial pathogens use to outcompete its rivals including molecules called colicins. A Type 6 Secretion System (T6SS) was recently described as contributing to *E. coli* killing in *S. sonnei*.

**Methods:**

We used Bulk Phenotyping of Epidemiological Replicates (BPER) which combined bacterial Genome Wide Association Studies (bGWAS) and high throughput phenotyping on a collection of *S. sonnei* surveillance isolates to identify the genetic features associated with *E. coli* killing and explore their relationship with epidemiological behaviour. We further explored the presence of colicins and T6SS components in the isolates using genomics, laboratory experimentation, and proteomics.

**Findings:**

Our bGWAS analysis returned known and novel colicin and colicin related genes as significantly associated with *E. coli* killing. *In silico* analyses identified key colicin clusters responsible for the killing phenotype associated with epidemiologically successful sub-lineages. The killing phenotype was not associated with the presence of a T6SS. Laboratory analyses confirmed the presence of the key colicin clusters and that killing was contact-independent.

**Interpretation:**

Colicins are responsible for *E. coli* killing by *S. sonnei*, not a T6SS. This phenotype contributes to shaping the observed epidemiology of *S. sonnei* and may contribute to its increasing prevalence globally. BPER is an epidemiologically relevant approach to phenotypic testing that enables the rapid identification of genetic drivers of phenotypic changes, and assessment of their relevance to epidemiology in natural settings.

**Funding:**

10.13039/501100000268Biotechnology and Biological Sciences Research Council, Biotechnology and Biological Sciences Research Council Doctoral Training Partnership studentship, 10.13039/100010269Wellcome Trust, 10.13039/501100000265Medical Research Council (UK), 10.13039/501100001665French National Research Agency.

**Research in context**

**Evidence before this study**

*Shigella sonnei* causes the acute diarrhoeal disease shigellosis and is increasing globally in prevalence for reasons which are not fully understood. As there is competition among inhabitants of the human gut for establishment, mechanisms for interbacterial competition are crucial for survival, however their role in an epidemiological context is poorly characterised. Colicins are a known mechanism of interbacterial competition among Enterobacteriaceae and have also been described in *Shigella* sp. An alternative competition mechanism, a Type 6 Secretion System was also recently described in an *S. sonnei* organism and was suggested to contribute to its epidemiological success. Identifying key determinants of interbacterial competition in, and their relationship with the epidemiology of, globally important diarrhoeal pathogens is thus a significant knowledge gap that requires innovative approaches.

**Added value of this study**

Our study identified the genetic determinants of *E. coli* killing in an unbiased way (bacterial Genome Wide Association Studies) and revealed multiple known and novel colicin and colicin-related genes. Contrastingly we found no association of the phenotype with T6SS configuration. We further identify the key colicin clusters that are responsible for killing in *S. sonnei* among an epidemiologically characterised collection of surveillance isolates. We show that epidemiologically successful sub-lineages contained colicins which were expressed extracellularly and mediated contact independent killing.

**Implications of all the available evidence**

Our study demonstrates the importance of colicins in mediating epidemiologically relevant interbacterial competition in *S. sonnei* and refutes the importance of a T6SS in this system. We have further identified several novel apparently colicin-related genes for further functional investigation. More broadly, our study framework of using ‘epidemiological replicates’ represents a move away from model organism approaches where combined high throughput phenotyping, genomic, and laboratory, analyses can rapidly identify genetic determinants of high epidemiological relevance in bacterial populations.

## Introduction

*Shigella* sp. cause >125 million cases of shigellosis that result in ∼212,000 deaths annually and understanding the biological drivers of its success as a pathogen is critical to public health.^1^^,^^2^ *S. sonnei* accounts for 24% of cases of shigellosis worldwide and contributes proportionately more to the disease burden in industrialising and high-income countries (HICs) where it is primarily transmitted through travel and among certain risk groups e.g., men who have sex with men (MSM).^3^, ^4^, ^5^ Recent genomic epidemiology studies of *S. sonnei* have revealed a concerning emergence of highly, and extensively drug resistant strains^6^, ^7^, ^8^, ^9^, ^10^, ^11^, ^12^, ^13^ leading to it being designated a WHO priority organism for AMR.^14^ While global subtyping systems can help us trace the shifting population dynamics, there is a need to further leverage these genomic epidemiology studies to better understand the biology of the organism and identify what drives certain sublineages to success.^15^

An example relevant to this study is the existence of four co-circulating subclades of MSM-associated *S. sonnei* in the UK during 2008–2014, three of which endured in time and spread internationally, and one of which did not [^16^ and see further details below]. Being an enteric pathogen, *S. sonnei* is in constant competition with the other members of the gut microbiota which acts as the barrier to successful infection and invasion of the gut epithelial cells in the colon.^17^ Interbacterial competition mechanisms are thought to be important for a multitude of bacterial pathogens such as *Burkholderia cepacia*, *Vibrio cholerae*, *Klebsiella pneumoniae*, and *Salmonella* spp.^18^, ^19^, ^20^, ^21^, ^22^ Therefore, mechanisms that are advantageous for interbacterial competition are likely to be beneficial for *S. sonnei*.

There is a plethora of interbacterial competitions mechanisms described, including colicins and Type VI secretion systems (T6SSs); the latter of which was recently described in *S. sonnei*.^23^^,^^24^ T6SSs are specialised secretion systems found in Gram negative bacteria capable of delivering a wide variety of effectors (usually antibacterial proteins) directly into the target cells.^25^^,^^26^ T6SSs function via a contractile sheath that propels a needle-like structure containing the spike complex with the effector protein into the adjacent target cell in a contact-dependent manner.^27^ In contrast to T6SS, colicins are not contact-dependent as they are secreted into the surrounding environment and translocated through the outer membrane of target cells by parasitising the existing protein translocation systems that perform other important biological functions in the sensitive cells.^28^ These toxic proteins are produced by many enteric bacteria and are usually encoded on a plasmid alongside the colicin lysis protein that is responsible for colicin release and the immunity protein that protects the host from its own colicins.^29^^,^^30^ Colicins have been studied extensively for more than three decades as a major component of inter-bacterial warfare with varying modes of action.^31^, ^32^, ^33^ Being a contact-independent process, production, and secretion of colicins with respect to complex bacterial spatial structures has been a focus of study in the recent years and how it depends on sensing and responding to the surroundings.^34^^,^^35^ While the importance of colicins for interbacterial competition/persistence have been shown *in vivo* with reports of colicins promoting diversity of the gut microbiota,^36^, ^37^, ^38^ the impact of colicins on bacterial population structures in real world settings remain poorly explored.

Here we sought to identify the mechanisms in *S. sonnei* responsible for interbacterial competition with a relevant microbiota competitor, *Escherichia coli,* and relate this to established epidemiological understandings. To do so, we leveraged a collection of sequenced isolates from a previous cross-sectional genomic epidemiology study of national surveillance in the United Kingdom from 2008 to 2014.^16^ As we were interested in comparing the behaviour of epidemiological sublineages, we conducted our experimental replicates at an epidemiologically relevant level (i.e., testing many clinical isolates from each sublineage rather than testing fewer representatives multiple times) in an experimental framework we call Bulk Phenotyping of Epidemiological Replicates (BPER). We advocate BPER as a concept for moving on from extrapolating from model organisms in the genomic era while also overcoming some of the complications of using poorly characterised clinical isolates for laboratory studies. By coupling BPER to bacterial Genome Wide Association Studies (bGWAS), we demonstrate that *E. coli* killing in *S. sonnei* is mediated by colicins found in epidemiologically successful sublineages.

## Methods

### Strains, their whole genome sequences, and phylogenetic tree construction

The main collection of *S. sonnei* genomes (n = 164 isolates, Table 1) used in this study originated from the archive of isolates from the national reference laboratory of UK Health Security Agency collected from 2008 to 2014 and has been described before.^16^ Sequencing data for all isolates is deposited in the European Nucleotide Archive and individual accession numbers are provided in Supplementary Table S1. An additional isolate, CIP 106374, which was also previously described was included in the bioinformatic analyses and sequenced as mentioned in Supplementary appendix along with the full details of phylogenetic tree construction and genotyping.

**Table 1:** Population structure summary of *S. sonnei* genotypes (n = 164) in this study.^b^

Genotype	Isolates (N)	Name	Original name	Epidemiological summary	Reference
3.6.1	10	CipR_parent	–	Subclade from which ciprofloxacin-resistant sublineage emerged	^15^
3.6.1.1	16	CipR	Ciprofloxacin-resistant Pop2	Triple QRDR^a^ mutation ciprofloxacin-resistant sublineage	^39^^,^^40^
3.6.1.1.3	4	–	–	Ciprofloxacin-resistant	^39^^,^^41^
3.6.1.1.3.1	7	CipR MSM1	MSM Clade 1	MSM-linked ciprofloxacin resistant isolates	^41^
3.6.2	9	Central Asia III Subclade	–	Associated with areas in Central Asia	^41^^,^^42^
3.6.3	9	Central Asia III Subclade	–	Associated with areas in Central Asia	^39^^,^^41^
3.7.16	7	Global III Subclade	–	–	^43^
3.7.18	19	Global III Subclade	MSM Clade 3	MSM-associated	^44^
3.7.25	46	MSM4	MSM Clade 4	MSM-associated	^41^
3.7.29.1.2	7	VN2 MSM2	MSM Clade 2	MSM-associated. Emerging from sweep 2 of Vietnam clone	^7^^,^^45^
3.7.30.4.1	6	OJC	OJC-associated	Associated with the Orthodox Jewish communities in Israel, UK, USA and Europe	^42^

a QRDR = quinolone resistance determining region.

b For brevity, only genotypes that included >4% of isolates are included in the table. Full genotyping data is available in Supplementary Table S1.

In addition to *S. sonnei,* several *E. coli* strains were used in this study including the laboratory strain MG1655, and three diverse isolates received at the National Reference Laboratory during 2023: including a commensal ST10 (O15:H16), an ST6199 (O1:H7), and a pathogenic ST131 (O25:H4) (Table 2).

**Table 2:** Summary of bacterial isolates (n = 163) used in this study and their origins.

Strains	Isolates (N)	Description	Reference
*S. sonnei*^a^	159	Cross-sectional isolates from routine surveillance during 2008–2014 collected by Public Health England (now UKHSA).	^16^
*S. sonnei* CIP 106347	1	*S. sonnei* clinical isolate from the collection at Institut Pasteur, previously described as having a functional T6SS	^24^
*E. coli* MG1655	1	*E. coli* MG1655 strain with a chromosomally encoded, constitutively expressed GFP marker and a kanamycin resistance marker	Previously used in^46^
*E. coli* LAES2471692004	1	Clinical isolate of *E. coli* belonging to ST6199 (O1:H7) received at the National Reference Laboratory	This study
*E. coli* LAE3140732004	1	Clinical isolated of *E. coli* belonging to ST131 (O25:H4) received at the National Reference Laboratory	This study
*E. coli* LAE31441672004	1	Clinical isolate of *E. coli* belonging to ST10 (O15:H16) received at the National Reference Laboratory	This study

a Various—see Supplementary Table S1 n = 159 as five isolates (compared with Table 1) failed to grow.

### Growth conditions for bacterial strains

All bacterial strains were grown in tryptone soy broth (TSB) (Millipore Sigma, UK) at 37 °C in a shaking incubator (220 rpm) unless otherwise stated. Antibiotic selection where applicable was carried out using kanamycin or azithromycin both at 30 μg/ml.

### Initial screen of subclade representatives for killing

Overnight pre-cultures of both *S. sonnei* and *E. coli* were diluted 1:100 (v/v) in fresh TSB and grown to mid-log phase. 10 μl of competition mixture containing *E. coli*: *S. sonnei* (1:10) was spotted onto a nitrocellulose membrane on a tryptone soy agar plate and incubated overnight at 37 °C. Competition mixture was then washed off into 500 μl of sterile PBS, serially diluted and plated on selective media for CFU enumeration.

### BPER using cell sorter

Culturable *S. sonnei* isolates corresponding to genomes (n = 159) were grown overnight in 96 well flat bottom plates (Greiner Bio One, UK) containing 150 μl of TSB and diluted 1:100 (v/v) into fresh TSB and grown for 2 h. *E. coli* was grown and diluted into fresh media as was done for initial killing assays and 880 μl of mid-log phase culture was diluted in 52 ml of TSB and 130 μl of that was distributed into each well of the 96-well plate. 20 μl of the mid-log phase *S. sonnei* cultures were then added to each well to make up a final volume of 150 μl with 1:10 (*E. coli*: *S. sonnei*) of the competition mixture and incubated overnight. The overnight competition mixtures were then diluted and GFP expressing *E. coli* cells which had survived the competition with *S. sonnei* were counted using a Bio-Rad ZE5 Cell Analyzer in a total of 10,000 events per well. The percentage of GFP expressing cells were calculated using FCS Express version 7 (De Novo Software) and plotted onto the phylogenetic tree using iTOL.^47^

### BPER growth assays using plate reader

Competition mixtures were set up similar to the cell sorter method albeit in a black 96 well plate (Greiner Bio One, UK). Fluorescence signal from the *E. coli* in the completion was measured every 15 min using a Synergy H1 multi-mode plate reader (BioTek Instruments) and a clear GFP signal corresponding to the optical density—based growth curve for *E. coli* was considered *E. coli* survival and used as a binary phenotype for *E. coli* killing.

### Genome wide association study

Draft assemblies of all isolates were assembled using Unicycler v0.4.8^48^ and annotated using Prokka v1.14.6.^49^ To generate the input for the GWAS kmer analysis, kmers were counted from assemblies using fsm-lite v1.0. Lastly, to generate the input file for the GWAS COG analysis, Roary v1.007002 was utilised to generate a gene presence/absence Rtab file containing the presence or absence of each gene in each isolate.^50^

After generation of appropriate inputs, GWAS was carried out using Pyseer v1.3.6.^51^ Pyseer uses linear models with fixed or mixed effects to estimate the effect of genetic variation in a bacterial population on a phenotype of interest, while accounting for potential confounding by population structure. For this investigation, presence or absence of the *E. coli* killing phenotype was utilised as the categorical phenotype (Supplementary Table S1). To account for population structure, all analyses were supplemented with phylogenetic distances from the mid-point rooted core genome phylogeny (above). Pyseer analyses were run using the linear mixed model (LMM). Specifically, the COG analyses were run using the gene presence absence file generated from Roary and the phylogenetic distances from the mid-point rooted core genome phylogeny. The default filters of Pyseer were utilised to filter out very low frequency COGs. Further GWAS investigations were carried out using Scoary v1.6.16^52^ utilising the gene presence/absence.csv file generated via the Roary pangenome pipeline as well as a trait file consisting of the *E. coli* killing phenotype for each sample.

### Colicin database construction and detection

To investigate the presence or absence of specific colicins within the isolates, a large quantity of colicin sequences were collated. Over 10,000 colicins from over 50 species of bacteria were collated from the European Nucleotide Archive as well as including some isolates from previously published sources.^53^ A multi-FASTA file containing the collated colicin sequences was utilised to generate a custom database via the prepareref command of ARIBA v2.14.6 where prepareref removes erroneous data and runs cd-hit to cluster the sequences based on a user-defined similarity threshold (90% in our case). ARIBA v2.14.6 was then run with the FASTQ files of all isolates, a percentage sequence ID cut-off of 95% and the colicin database to report which sequences were observed in each isolate. The *E. coli* isolates were screened for the colicins and colicin related genes using ABRicate v1.0.1 (https://github.com/tseemann/abricate) under the default settings using our custom colicin database. The database of colicin sequences are publicly available through figshare (10.6084/m9.figshare.20768260.v1).

### Statistical testing

All statistical analyses were performed using R v3.6.1. To determine the significant difference between the T6SS profiles of the killing versus the non-killing isolates, an independent t-test was undertaken comparing the number of T6SS elements in each group (i.e., killing and non-killing). Further independent t-test were undertaken to determine the association of the killing phenotype with each detected colicin cluster to determine their order of iterative addition as ‘key colicin clusters’ (see Results).

### Exploring the presence of the T6SS

The 18 components of the full T6SS were extracted from the annotated Nanopore sequence of *S. sonnei* CIP 106347 and compiled into a singular multi-FASTA file. SRST2 v0.2.0^54^ was then utilised for the purpose of T6SS component gene detection as a custom database. The T6SS components within the multi-FASTA were not pre-clustered and therefore were assigned to gene clusters based on 90% nucleotide similarity via CD-HIT v4.8.1.^55^ Python scripts provided as part of the SRST2 package were utilised to parse the clusters and generate a SRST2 compatible database. SRST2 gene detection was then run utilising the custom T6SS database.

### Role of funders

Funders did not have any role in study design, data collection, data analyses, interpretation, or writing of report.

## Results

### The epidemiology and global context of the isolate collection

**Fig. 1:**
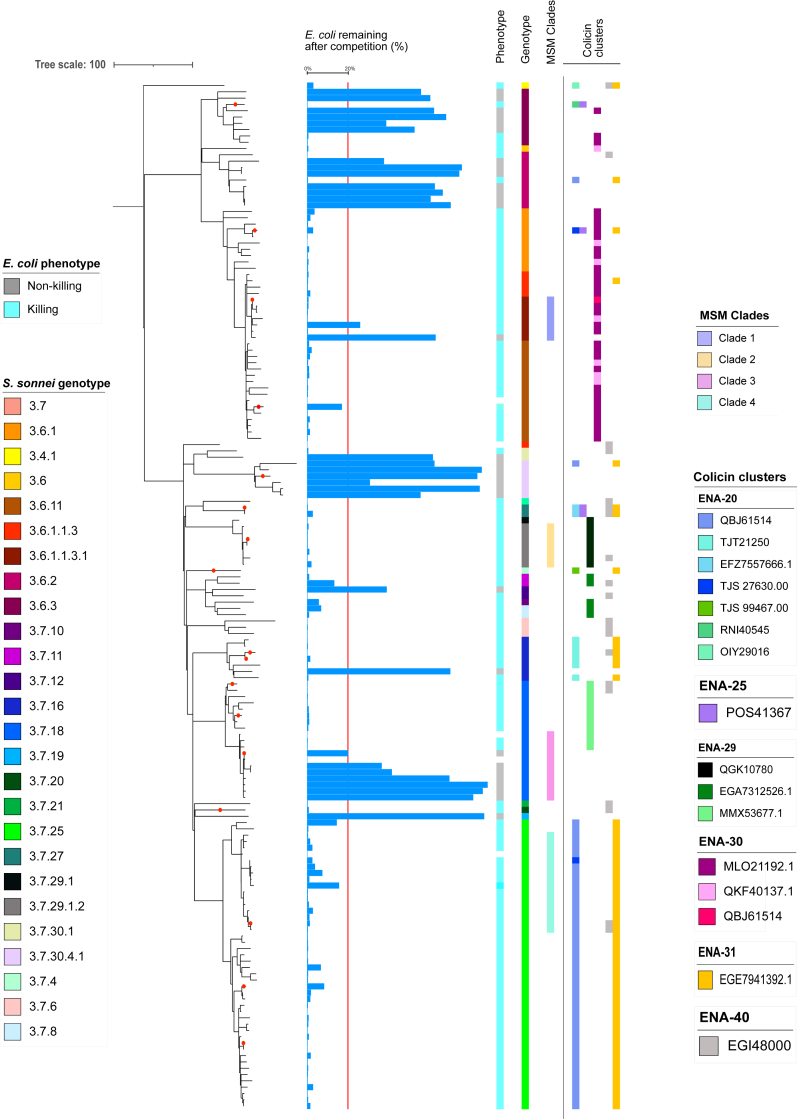
**The distribution of *E. coli* killing activity and colicin clusters associated with the phenotype across genotypically diverse Lineage 3 *S. sonnei***. The tree is a rooted maximum likelihood phylogeny for 165 *S. sonnei* Lineage 3 isolates. Red circles overlaying terminal branches/nodes indicate isolates which were utilised for colicin assay and mass spectrometry. The horizontal blue bars show a continuous measure of *E. coli* killing (GFP%–see methods) with a vertical red line highlighting a cut off (≤20%) for *E. coli* killing. A binary killing/non-killing phenotype determined by plate reading is depicted in the colour strip closest to the tree followed by *S. sonnei* genotype, assignment to co-circulating MSM clades, and key colicins clusters associated with the killing phenotype. Individual colicins are shown by different colours labelled, by their European Nucleotide Archive accession numbers) and are grouped (and in columns) by arbitrarily named genetic clusters generated by the ARIBA package (see methods and Supplementary Table S2).

A collection of *S. sonnei* genomes used in this study came from a cross sectional subsample of routine microbiological surveillance in the United Kingdom from between 2008 and 2014 (n = 164).^16^ The original epidemiological study revealed the presence of four distinct clades which were co-circulating among MSM. In this study, Clades 1, 2, and 4 showed markers of population level epidemiological success (specifically international spread and prolonged circulation) relative to Clade 3, which was not successful.^16^ Notably, a recently described international global genotyping framework has renamed these Clades 1–4 (above) as Subclades 3.6.1.1.3.1 (CipR.MSM1), 3.7.29.1.2 (VN2.MSM2), 3.7.18, and 3.7.25 (MSM4) respectively.^15^ These isolates were utilised along with CIP 106347, which was previously used in studies of a putative *S. sonnei* T6SS^24^ to construct a detailed phylogeny complemented with genotype assignations (Fig. 1).

All *S. sonnei* belonged to the globally disseminated multidrug resistant lineage of *S. sonnei*, Lineage 3 (Table 1, Fig. 1). Further genotyping showed that most isolates (61%, n = 101) belonged to Clade 3.7, particularly Subclade 3.7.25 (MSM4, 28%, n = 46) and Subclade 3.7.18 (12%, n = 19) (Table 1). The remaining isolates (39%, n = 64) belonged to Subclade 3.4.1 (Latin American III) and various Subclades of Clade 3.6 (Central Asia III) including 3.6.1 (CipR_parent), 3.6.1.1 (CipR), 3.6.1.1.3, 3.6.1.1.3.1 (CipR MSM1), 3.6.2, and 3.6.3 (Table 1). Correlating the genotype names with epidemiological history revealed that various globally important subclades of *S. sonnei* were captured in our dataset including internationally disseminating antimicrobial resistant genotypes (Table 1 and Supplementary Table S1). Thus, our collection contained a breadth of the diversity of the globally disseminated Lineage 3.

### *E. coli* killing is common and associated with genotype in *S. sonnei*

After an initial low throughput screen revealed differences in the *E. coli* killing phenotype among Subclade representatives (Supplementary Figure S1), we scaled our approach to include the entire collection of *S. sonnei*. We did this so our laboratory assays were being replicated at our desired level of inference i.e., we wanted to relate the phenotype to the epidemiological behaviour of different genomic subtypes, so we needed multiple clinical isolates that belonged to those subtypes, rather than biological or technical replicates (though for robustness we also included duplicate technical replicates), which we call BPER.

Owing to the number of isolates involved and our ambition to scale the BPER approach further in future studies, we assayed the killing phenotype in a high-throughput manner. Specifically, we used cell sorting to measure the proportion (%) of green-fluorescent *E. coli* remaining after overnight competition with *S. sonnei* in a 96-well format. As a cell sorter is a specialist equipment, we also correlated these results with a binary read out of green fluorescence following a simple plate-reader based growth assay which is more broadly available. Introducing a threshold of positivity (20%) among the cell sorting results of these assays led to 99% concordance between the two approaches (Fig. 1, Supplementary Table S1, see methods). We found that most 82% (131/159) of *S. sonnei* displayed *E. coli* killing and that phenotype clustered with genotype (Fig. 1). The continuous data (from cell sorting) revealed a spectrum of difference in the phenotype with the proportion of *E. coli* following competition being between 0% and 89% (mean = 14%). Associating the phenotype against the population structure of *S. sonnei* revealed that Subclades of the Central Asia III Clade (3.6.3 and 3.6.2), 3.7.18 (MSM Clade 3), and 3.7.30.4.1 (a Subclade associated with transmission among Orthodox Jewish Communities) were predominately non-killing with the remaining Subclades being predominately killing (Fig. 1). However, differences among isolates belonging to the same Subclade (i.e., epidemiological replicates) demonstrated phenotypic variation (e.g., genotype 3.7.18 where isolates ranged from 0.1% to 88.44% with a mean of 25.15%), highlighting the value of the BPER approach when assaying clinical isolates for inference at an epidemiological level.

To further explore the relevance of this phenotype for *S. sonnei* in the real world, we also determined killing phenotypes of the *S. sonnei* collection against clinical *E. coli* isolates, including both commensal and other pathogenic types. As expected, clinical isolates of *E. coli* exhibited greater levels of survival from the *S. sonnei* competitors (Supplementary Figure S3, Supplementary Table S1). Interestingly, the degree of *E. coli* killing by the *S. sonnei* strains used scaled intuitively with the nature of the *E. coli* strain (i.e., killing of lab strain > commensal strain > pathogenic strains) and the colicin and colicin-related protein content of the *E. coli* strains (see below). Specifically, and critically for the translatability of these results, the commensal Multi Locus Sequence Type 10 (ST10) was killed by a greater proportion of *S. sonnei* strains than the other, more pathogenic sequence types (STs) (55/159 (35% 95% Confidence Interval [CI] 23%–43%) compared with 10% (16/159, 95% CI 4–24%) for ST6199 and 8% (12/159, 95% CI 7–23%) for ST131, Supplementary Figure S3, Supplementary Table S1).

### GWAS indicates that colicins are responsible for *E. coli* killing in *S. sonnei*

To identify genetic factors responsible for *E. coli* killing, we conducted bGWAS for association with the *E. coli* killing phenotype (using pyseer and MG1655, see methods) and focused on those results that related to predicted/annotated genes. This revealed a variety of genetic factors that were significantly positively associated with the *E. coli* killing phenotype including 3187 kmers within 22 genes (short sequence fragments of length k = 10–100, maxp>15) and 64 clustered orthologous groups (COGs) (lrt p-value <0.05) (Supplementary Table S2).

**Fig. 2:**
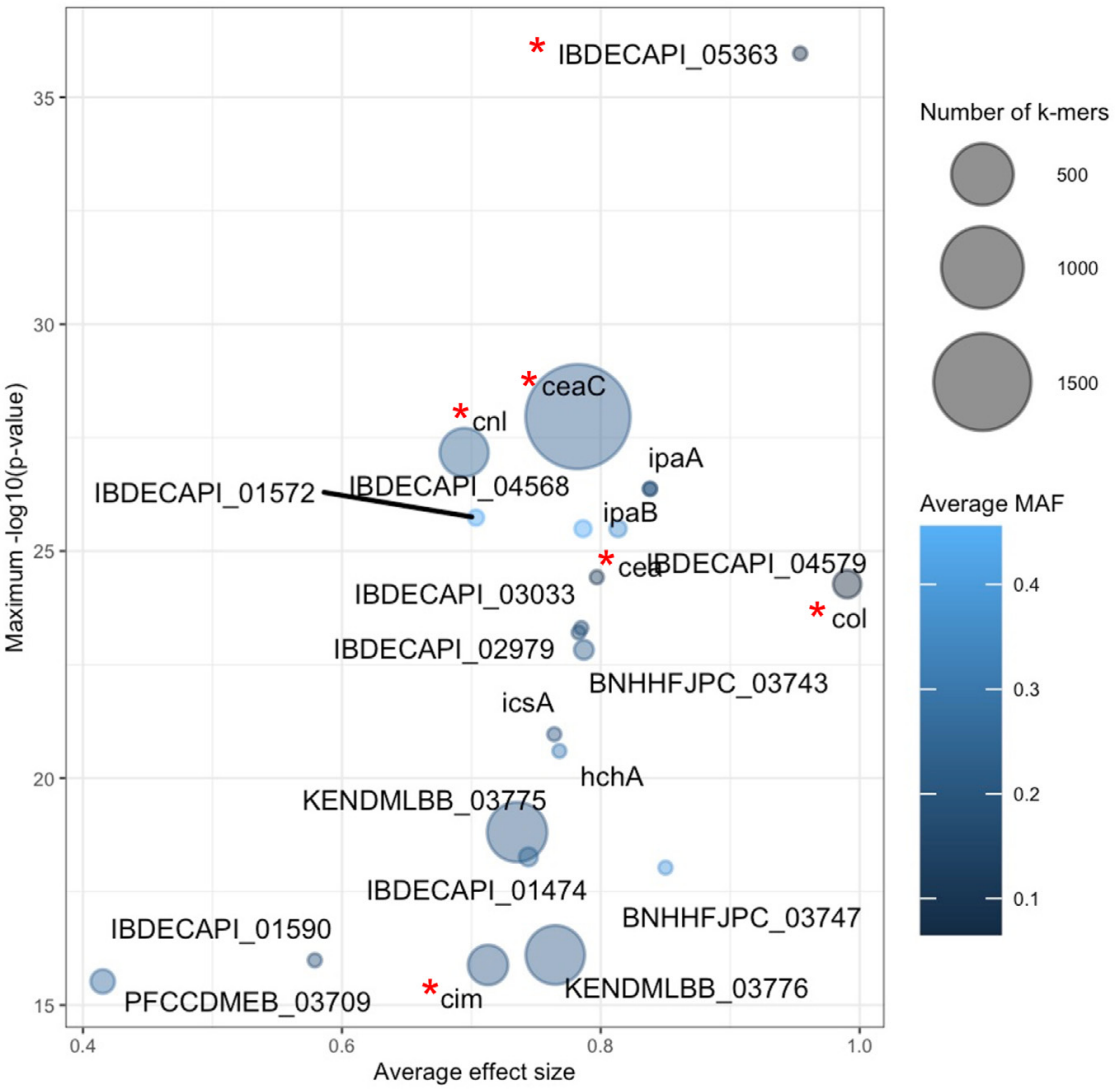
**Genes associated with *E. coli* killing across *S. sonnei* (including measures of association)**. The bubble plot shows the genes (text labels in graph field) associated with the *E. coli* killing phenotype. Each gene is represented by a bubble scaled in size by the number of sequence elements (kmers) contained within the gene that were associated with the killing phenotype. The position on the axes captures the effect size (x-axis) and statistical support (y-axis as negative logarithm of the p-value) for the association with the killing phenotype. The bubbles are coloured according to the average Minor Allele Frequency (MAF) of the kmers relating to the gene according to the inlaid heat map. Red stars indicate those genes with colicin or colicin-related function.

Investigating the 22 genes which contained kmers significantly associated with killing revealed one hypothetical protein encoding gene and 21 of known function; six of which were colicin related. These were IBDECAPI_05363, *cim*, *cnl*, *cea*, *ceaC* and *col* (Fig. 2, Supplementary Table S2). IBDECAPI_05363 was the strongest result (i.e., had the highest effect size and lowest p-value, see Fig. 2) and displayed 99% similarity to the *E. coli* E1 colicin immunity protein. The *cim* gene was originally described on the *E. coli* plasmid CloDF13 and is known to be bacteriocinogenic and *cnl* encodes a lysis protein for colicin N originally described as encoded on the *E. coli* plasmid pCHAP4.^56^^,^^57^ The genes *cea*, *col* and *ceaC* encode for the colicins E1, E2 and E3 respectively and were originally identified on small plasmids ColE1, ColE2 and ColE3.^58^^,^^59^ In further support of the contribution of colicins to the killing phenotype, two of these genes (*cnl* and *ceaC*) were the first and ninth best supported genes in the COG analysis, and a COG analysis using an alternative GWAS approach (scoary, see methods) also identified the colicin related genes *imm* and *cnl* as strongly associated with the *E. coli* killing phenotype (Supplementary Table S2). Owing to the significant burden of evidence that colicins were responsible for the *E. coli* killing phenotype, we explored the distribution of these proteins further (see below).

Genes containing kmers positively associated with the killing phenotype that were not obviously related to colicins included phage related genes (n = 4), T3SS related genes (n = 3), DNA modification/binding genes (n = 2), protein deglycase gene (n = 1) and plasmid replication/transfer genes (n = 5) (Supplementary Table S2). Notably, the plasmid replication/transfer genes from the kmer analysis (KENDMLBB_03776, BNHHFJPC_03747, PFCCDMEB_03709, BNHHFJPC_03743 and IBDECAPI_04568) encoded for replication protein B (IBDECAPI_04568), repE (BNHHFJPC_03743) with the latter (RepE protein) also being present in the COG analysis. Replication protein B and RepE have been shown to initiate replication of both chromosomal segments and plasmids within *E. coli* highlighting its potential for initiation of replication of small plasmids, such as those encoding colicins.^60^ It is possible that these plasmid-related genes were associated to the killing phenotype by virtue of their co-location with colicin-related genes.

### Various colicins are widely distributed in *S. sonnei*

To determine the distribution of colicins in *S. sonnei* we screened the genomes against a custom database of >10,000 colicin sequences that grouped into 145 clusters (methods). Although this revealed that widespread distribution of colicins (all isolates contained ≥1 colicin), no single colicin or cluster showed perfect concordance with the presence of the killing phenotype across *S*. *sonnei* (Supplementary Table S1, Fig. 1), suggesting no single protein was responsible for the phenotype. To determine which colicin clusters were most likely responsible for the killing phenotype, we determined the association of colicin clusters with the binary *E. coli* killing phenotype and ordered cluster inclusion based on statistical support for the association with the phenotype (i.e., starting with the lowest p-value first) with each cluster being added until all *E. coli* killing could be explained without clusters overlapping with the non-killing phenotype (Fig. 1). This process resulted in six key clusters being identified that explain the distribution of the *E. coli* killing in *S. sonnei*.

To further validate the association of colicins with the *E. coli* killing phenotype, we selected 15 isolates representative of the breadth of the key colicin clusters distributed throughout the phylogeny and two isolates that did not display *E. coli* killing as controls (Fig. 1). We then extracted culture supernatants of these isolates (n = 17) to confirm the presence of the colicins in the supernatant. Laboratory assay for contact-independent killing confirmed that filtered supernatants from the 15 colicin cluster-containing *S. sonnei* killed *E. coli* MG1655 while those from the two non-killing isolates did not (Supplementary Figure S2). Mass spectrometry on filtered supernatants (details in Supplementary Appendix) from all isolates (100%, 15 of 15) that displayed *E. coli* killing contained at least one contributing colicin in the total peptides identified in the samples and, for 87% (13 of 15) of isolates, the proteins matched to a unique peptide sequence of an individual colicin (Supplementary Table S3). Mass spectrometry also confirmed that, the supernatants from the two isolates that did not display *E. coli* killing did not yield any matches to these colicin sequences further confirming the role of colicins in *E. coli* killing (Supplementary Table S3).

To interpret our varied killing results across *E. coli* strains we also screened these strains against the colicin database. The screened genomes of the *E. coli* challenge strains revealed that while the MG1655 and ST10 strains both contained only two (and identical) colicin and colicin related proteins, the ST6199 and ST131 strains contained an additional five proteins each (Supplementary Table S6, see below and methods).

### *E. coli* killing *in vitro* in *S. sonnei* is not mediated by T6SS

**Fig. 3:**
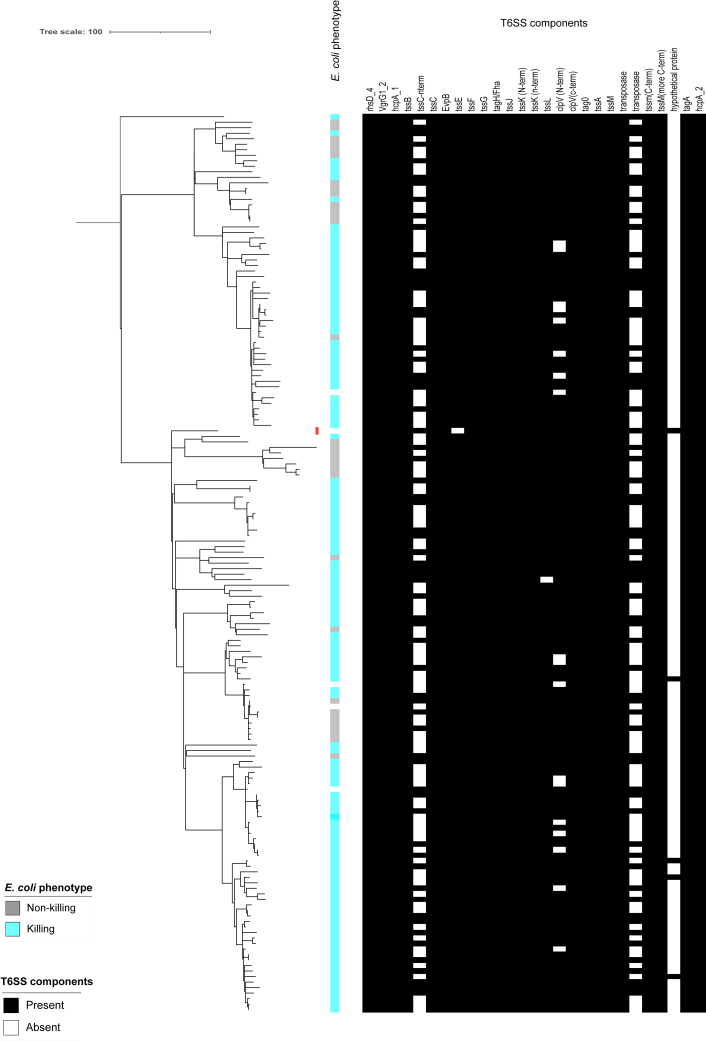
**Genotypic T6SS profiles across Lineage 3 *S. sonnei***. The tree is a rooted maximum likelihood phylogeny for 165 *S. sonnei* Lineage 3 isolates. A red colour block is shown to the right of the CIP 106347 isolate (in which the T6SS was previously described). The killing/non-killing phenotype is depicted in the colour strip closest to the tree followed by colour strips depicting the presence/absence of individual T6SS components, both coloured according to the inlaid keys.

Our results highlight the critical role of colicins in mediating *in vitro E. coli* killing in *S. sonnei*. As a previous study described a functional T6SS in *S. sonnei* clinical isolate CIP 106347 that was hypothesised to contribute to competition with *E. coli* and *S. flexneri,* we also explored the possible role of the putative T6SS in *E. coli* killing.^24^ To do this we re-sequenced CIP106347 and extracted the gene sequences for predicted proteins in the region of the T6SS system. Predicted proteins were used as the reconstituted locus was inconsistent with that originally described^24^ and contained multiple interruptions that would render the apparatus non-functional (Supplementary Figure S4). The assembly is available in GenBank under accession number CP109775.1 and the T6SS locus is at coordinates 3535861–3560362. In fact, one or more of the key components of the T6SS (e.g., N terminus of TssC) were absent in 100% of our isolates and in CIP 106347, suggesting that the T6SS in *S. sonnei* is likely non-functional (Fig. 3). Consistent with this suggestion is the lack of a correlation between the presence and absence of components of the CIP 106347 T6SS locus and the killing phenotype in our studies (Fig. 3), though notably our liquid media experimental model is insufficient to evaluate a functional T6SS. Thus, the T6SS genotypic profiles suggest that T6SS is not responsible for our phenotype and is likely non-functional across Lineage 3 *S. sonnei*.

## Discussion

Outcompeting commensals like *E. coli* in the gut is an important aspect for *Shigella* for surviving and establishing in their niche. Since *Shigella* is known to have a relatively lower infectious dose, we can expect that the mechanisms by which *Shigella* establishes itself are relatively efficient.^61^ Therefore, it is important to investigate these mechanisms to better understand the dynamics of the complex gut microbial communities that lead to infection, and correlate this with established epidemiological understandings. While there will be several mechanisms at play in the complex environments and microbial communities that the pathogens live in, we demonstrated here the crucial role colicins play in interbacterial competition with *E. coli* and the potential consequences of their actions at an epidemiological level.^62^^,^^63^ The important role of colicins in the genus *Shigella* could be further evidenced by recent reports of colicins being discovered in *S. flexneri* 2a^64^ and a previous description of colicin plasmid acquisition in establishing *S. sonnei* in Vietnam.^45^ Our findings strengthen this evidence base by our use of a diverse collection of real-world isolates and assay of the phenotype with replication at an epidemiologically relevant level through BPER.

The high prevalence of *E. coli* killing throughout the *S. sonnei* phylogeny suggests that this is an epidemiologically important phenotype. This is further supported by the only non-killing Subclade (3.7.18, Global III subclade (MSM-associated Clade 3)) being outcompeted by *E. coli* killing Subclades (CipR MSM1 (3.6.1.1.3.1), MSM4 (3.7.25), and VN2 MSM2 (3.7.29.1.2)) in a scenario where the Subclades were known to be co-circulating in a single patient population.^16^ Of course, phenotypes other than *E. coli* killing will also contribute to the competition dynamics among different genotypes. Indeed, our previous work identified that Subclade 3.7.18 was also comparatively distinct in lacking a low fitness cost azithromycin resistance plasmid, pKSR100.^16^^,^^46^ Non-killing phenotypes were also conserved in other Subclades of Central Asia III (3.6.2 and 3.6.3) and 3.7.40.1 (Global III OJC). For the majority of Lineage 3 clades, the *E. coli* killing ability was conserved through clade expansion indicating its potential to contribute to the global success of Lineage 3 *S. sonnei*. However, it is important to consider the real-world relevance of our findings. Our extended BPER experiments with other *E. coli* competitors showed that the killing phenotype is less prominent in clinical isolates. This may be due to a variety of factors, including the presence of the O-antigen in the clinical isolates which has previously been shown reduce colicin activity.^65^ Importantly however, our killing phenotype was more concordant with killing of ST10, a common commensal ST which represents the type of *E. coli* most likely to be competing with *S. sonnei*. Bacteriocins have previously been shown to be important in the virulence of pathogenic extraintestinal *E. coli*^66^ and this is via a number of different mechanisms of actions for those colicins present.^30^ Consistent with this finding, *S. sonnei* were less able to kill the pathogenic ST6199 and ST131 *E. coli* strains, which contained more colicins and colicin immunity proteins than MG1655 and ST10. Hence, we have shown that colicins are the key driving factor responsible for *E. coli* killing with relevance to commensal strains.

We advocate the BPER approach as a way of assessing the phenotypes in an epidemiologically relevant way. Since most studies carry out laboratory experiments of phenotypes using either type strains or a limited number of clinical isolates, the level of applicability of what could occur in a real-world setting might not be fully captured. Therefore, we propose that using a collection of real-world isolates in laboratory phenotype experiments and having epidemiological replicates where possible rather than biological replicates offer greater insight into relevant pathogen biology. We found that, in the case of this phenotype, BPER could be implemented in a simple, cost-effective experimental setup with no/minimal compromise on accuracy (only two mismatches between the experiments using flow cytometry and plate reader, Supplementary Table S1). Here, by working with a real-world isolate collection with known epidemiological outcomes we could work backwards to identify the factors that helped shape the observed epidemiology and deepen our understanding of the biology in a targeted, comparatively rapid manner.

We have shown that *in vitro E. coli* killing by *S. sonnei* is common (although the killing percentage reduced with the use of clinically isolated *E. coli* strains possibly due to bacteriocins produced by those *E. coli* strains^67^) and likely mediated by colicins not via a T6SS, as previously suggested.^24^ This was supported by the lack of a fully intact T6SS in any of the isolates under study and no association of putative T6SS components with the killing phenotype. However, it is important to note that T6SS is a contact-dependent killing mechanism that would be more appropriately evaluated by *in vivo* experiments in animal models. However, this would be an inappropriate experiment to undertake with a large bank of strains, and unnecessary given that the genomic evidence suggests there are no intact T6SS. We did however find that our liquid media competition experiments matched the outcomes of our initial solid-media experiments for a subset of strains (Supplementary Figure S1, Supplementary Table S1), consistent with the killing phenotype being driven by colicins. Furthermore, this is well supported by associations between colicin clusters and the *E. coli* killing phenotype and confirmation of the secretion and functionality of colicins *in vitro*.

Excitingly, our work also identified several novel genes that were associated with the *E. coli* killing phenotype not known to be part of colicin synthesis or activity (i.e., four phage related genes and one hypothetical gene). These may have some currently unknown relationship with the colicins (e.g., phage proteins may be involved in the mobility of the colicin plasmids) or have as yet unknown functions. In any case, these genes represent ideal candidates for future studies investigating the mechanisms of interbacterial competition in *Shigella*. Furthermore, although, not practicable at the scale used in this study, mutation experiments with individual isolates are needed to definitively confirm (or reconfirm) the colicin-mediated killing (and/or immunity). However, previous work has shown that colicins produced by *S. sonnei* facilitate *E. coli* killing further supporting our findings.^68^ Ultimately testing mutants of colicins, their immunity proteins, and associated genes of unknown function in *in vivo* models represent be exciting avenues of future research to explore the mechanisms more fully. Meanwhile, at an epidemiological scale, our BPER approach has demonstrated the likely importance of interbacterial competition from a panel of diverse strains supporting the importance of this phenotype in shaping bacterial population dynamics.

## Contributors

P.M.D.S—Methodology, Formal analysis, Investigation, Writing—original draft, Writing—Review & editing, Visualisation, R.J.B—Methodology, Software, Formal analysis, Investigation, Writing—original draft, Writing—Review & editing, Visualisation.

L.K—Methodology, Formal analysis, Investigation, Data curation, Writing—Review & editing, Visualisation.

P.N—Methodology, Formal analysis, Investigation, Data curation, Writing—Review & editing, Visualisation.

L.D—Methodology, Writing—Review & editing.

E.N—Methodology, Formal analysis, Investigation, Data curation, Writing—Review & editing.

B.H—Conceptualisation, Supervision, Writing Review & editing, Supervision.

F-X.W—Conceptualisation, Resources, Writing—Review & editing, Supervision.

B.S.M− Conceptualisation, Resources, Writing—Review & editing, Supervision.

C.J—Conceptualisation, Resources, Writing—Review & editing.

K.S.B—Conceptualisation, Methodology, Formal analysis, Resources, Writing—original draft, Writing—Review & editing, Visualisation, Supervision, Funding acquisition.

## Data sharing statement

Sequencing data for all isolates are deposited in the European Nucleotide Archive and individual accession numbers are provided in Supplementary Table S1.

The colicin sequence database used in this study is publicly available through figshare via https://doi.org/10.6084/m9.figshare.20768260.v1.

Mass spectrometry data are deposited in the ProteomeXchange Consortium via the PRIDE partner repository with the dataset identifier PXD036656.

## Declaration of interests

Authors declare no competing interests.
