# Supplementary material for: Escherichia coli killing by epidemiologically successful sublineages of Shigella sonnei is mediated by colicins
Source: eBioMedicine. 2023 Oct 6;97:104822. doi: 10.1016/j.ebiom.2023.104822 (PMC10579285; doi:10.1016/j.ebiom.2023.104822)
Supplement: Supplementary Material [file mmc1.docx]

**Supplementary appendix for *Escherichia coli* killing by epidemiologically successful sublineages of *Shigella sonnei* is mediated by colicins**

**Authors and affiliations**

P. Malaka De Silva^*1^, Rebecca J. Bennett^*1^, Lauriane Kuhn^2^, Patryk Ngondo^3^, Lorine Debande^3^, Elisabeth Njamkepo^4^, Brian Ho^5^, François-Xavier Weill^4^, Benoît S. Marteyn^3^, Claire Jenkins^6^, Kate S. Baker^1^

^*^Authors contributed equally

^1^Department of Clinical Infection, Microbiology, and Immunology; Institute for Infection, Veterinary, and Ecological Sciences (IVES), University of Liverpool, Liverpool, United Kingdom

^2^ Plateforme protéomique Strasbourg Esplanade FR1589 du CNRS, Université de Strasbourg, Strasbourg, France

^3^ Université de Strasbourg, CNRS, Architecture et Réactivité de l’ARN, UPR9002, F-67000 Strasbourg, France

^4^Institut Pasteur, Université Paris Cité, Unité des Bactéries pathogènes entériques, Centre National de Référence des *Escherichia coli*, *Shigella* et *Salmonella*, Paris, F-75015, France

^5^Institute of Structural and Molecular Biology, University College London and Birkbeck, London, UK

^6^Gastro and Food Safety (One Health) Division, UK Health Security Agency, Colindale, London, UK

**Corresponding author**

Kate S Baker

kbaker@liverpool.ac.uk

**INDEX**

| Supplementary Methods …………………………………………………………………………. | 2 |
| --- | --- |
| Supplementary Figure 1 ………………………………………………………………………….. | 6 |
| Supplementary Figure 2 ………………………………………………………………………….. | 7 |
| Supplementary Figure 3 ………………………………………………………………………….. | 8 |
| Supplementary Figure 4 ………………………………………………………………………….. | 10 |
| Supplementary tables as separate files ………………………………………………………… | 11 |
| References ………………………………………………………………………………………… | 12 |

**Supplementary methods**

*Growth and sequencing of CIP 106347 strain*

The CIP 106347 strain was cultured overnight at 37 °C in alkaline nutrient agar (20 g casein meat peptone E2 from Organotechnie; 5 g sodium chloride from Sigma; 15 g Bacto agar from Difco; distilled water to 1 L; adjusted to pH 8.4; autoclaved at 121°C for 15 min). A few isolated colonies from the overnight culture were used to inoculate 20 mL of Brain-Heart-Infusion (BHI) broth and were cultured until a final OD_600_ of 0.8 was reached at 37°C with shaking (200 rpm —Thermo Scientific MaxQ 6800). The bacterial cells were harvested by centrifugation and the DNA extraction was performed by the used of Genomic-tip 100/G column (Qiagen) according to the manufacturer’s protocol. The library was prepared according to the instructions of the “Native barcoding genomic DNA (with EXP-NBD104 and SQK-LSK109) procedure provided by Oxford Nanopore Technology. Sequencing was then performed on a MinION Mk1C apparatus (Oxford Nanopore Technologies). The genomic sequences of the isolates were assembled from long and short reads, with a hybrid approach and UniCycler version 0.4.8 (1). A polishing step was performed with Pilon version 1.23 to generate a high-quality sequence composed of chromosomal and plasmid sequences (2). The sequencing data is deposited in NCBI under the BioProject accession number PRJNA890640. All metadata and sequence data accession numbers are provided in Supplementary Table 1.

*Phylogenetic tree construction*

FASTQ sequence files were mapped against the reference *S. sonnei* 2a strain 53G (HE616528.1) concatenated with its multiple plasmids (HE616529.1, HE616530.1, HE616531.1, HE616532.1) using Burrows-Wheeler Aligner (BWA) v0.5.9-r16 (3). The mapping files were filtered and sorted using samtools (4). Duplicates were marked using Picard tool. Subsequent variant calling was completed through bcftools and a consensus file was generated for each isolate (5). Each chromosome sequence was extracted, and regions were masked using a mask file containing plasmid sequences, IS elements and repeat regions. Gubbins v2.3.4 removed duplicate and low-quality sequences followed by SNP-sites to obtain the core-genome alignment (6, 7). RAxML-ng was then utilised to infer a phylogenetic tree (8). Each phylogenetic tree has been midpoint rooted with visualisations completed using interactive Tree of Life (iTOL) v6.1.1 (9).

*Genotyping*

Mykrobe v0.10.0 was utilised on the FASTQ sequences of all isolates (10). The output from Mykrobe was then parsed using a custom python script (<https://github.com/katholt/sonneityping/>) based on the genotyping scheme proposed by Hawkey *et al*., (2021) (11) . Upon parsing of the Mykrobe output, a tsv file containing the genotype was generated.

*Mass spectrometry analysis of supernatants and data post-processing*

Shigella sonnei secreted proteins from culture supernatants, precipitated with TCA (Trichloroacetic acid) were resolubilized in UTCT buffer (urea 2M, thiourea 7M, CHAPS 4%, Tris HCl 20 mM pH 7.8) for 1 hour at 30°C under shaking (800rpm) and clarified by centrifugation at 12 000 g at 4°C for 15 minutes. The samples were prepared for MS analysis as described in a previous study (12). Briefly, 10µg of each sample were precipitated with 0.1 M ammonium acetate in 100% methanol, and proteins were resuspended in 50 mM ammonium bicarbonate. After a reduction-alkylation step (dithiothreitol 5 mM – iodoacetamide 10 mM), proteins were digested overnight with 200ng of sequencing-grade porcine trypsin (Promega, Fitchburg, MA, USA). The resulting vacuum-dried peptides were resuspended in water containing 0.1% (v/v) formic acid (solvent A). The peptide mixtures were analyzed using a U3000-RSLC system (Thermo-Fisher Scientific, USA) coupled to a TripleTOF-5600 mass spectrometer (Sciex, Canada) operating in positive mode with a nanoelectrospray source. 500ng of each sample were loaded on a C-18 precolumn (75 μm ID × 20 mm nanoViper, 3µm Acclaim PepMap; Thermo-Fisher Scientific) at 2µL/min with solvent A during 10min. After desalting and concentration, the pre-column was switched online with the analytical C18 analytical column (75 μm ID × 25 cm nanoViper, 3µm Acclaim PepMap; Thermo-Fisher Scientific). Peptides were eluted by using a 5%-40% gradient of solvent B (0.1 % FA in Acetonitrile) for 250 minutes at a 300 nL/min flow rate. The TripleTOF 5600 was operated in high-sensitivity data-dependent acquisition mode with Analyst software (v1.8, ABSciex) on a 400-1250 m/z range. Survey MS scans were acquired during 250ms and up to 20 of the most intense multiply charged ions (2+ to 5+) were selected for CID fragmentation with a rolling collision energy during 60msec. A dynamic exclusion time of 15 s was applied during the peak selection process. The mass spectrometric data were deposited to the ProteomeXchange Consortium via the PRIDE partner repository with the dataset identifier PXD036656 (Supplementary Table 4).

*Database search and mass-spectrometry data post-processing*

Raw data were converted to Mascot Generic File format (.mgf) using msConvert software (ProteoWizard). MS peaklists were searched against the UniProtKB database from *S.sonnei* (release 2021_01, 22219 sequences) concatenated to a home-made database consisting of all *E.coli* colicins, immunity and lysis proteins. We used the Mascot algorithm (version 2.8.1, Matrix Science) to perform the database search with a target-decoy strategy. The resulting .dat Mascot files were then imported into Proline v2.1 package (13) for further post-processing. Proteins were validated on Mascot pretty rank equal to 1 and 1% FDR on peptide spectrum match (PSM) level. The relative protein abundance within a sample was estimated using the Spectral Count approach: the total number of MS/MS spectra was computed while considering shared and unique peptides (BASIC Spectral Count) or only unique peptides (SPECIFIC Spectral Count).

*Cell-free supernatant assay for E. coli killing*

All relevant *S. sonnei* strains were grown overnight in Tryptone Soy Broth (TSB) at 37˚C with 200 rpm shaking in individual tubes. After overnight growth 1ml of bacterial culture was filtered using a 0.22µm filter and the filtrate was collected in sterile Eppendorf tubes and used in the assay. For the *E. coli* killing assay, *E. coli* MG1655 was grown overnight in similar conditions to the *S. sonnei* cultures and were diluted 1:100 (v/v) in fresh medium and grown for 2 hours to reach exponential phase. These cultures were then diluted 1:10 in sterile PBS and 100µl of the diluted culture was added to 5ml of sterile PBS, which was then swabbed on a Tryptone Soy Agar plate using a sterile cotton swab. Sterile filter paper discs were then applied on to the freshly swabbed lawn of *E. coli* and 10µl of *S. sonnei* filtrates were spotted onto the filter paper discs and the plates were incubated without shaking at 37˚C overnight. The zones of inhibition around the discs were then photographed following incubation and shown in Supplementary Figure 2.


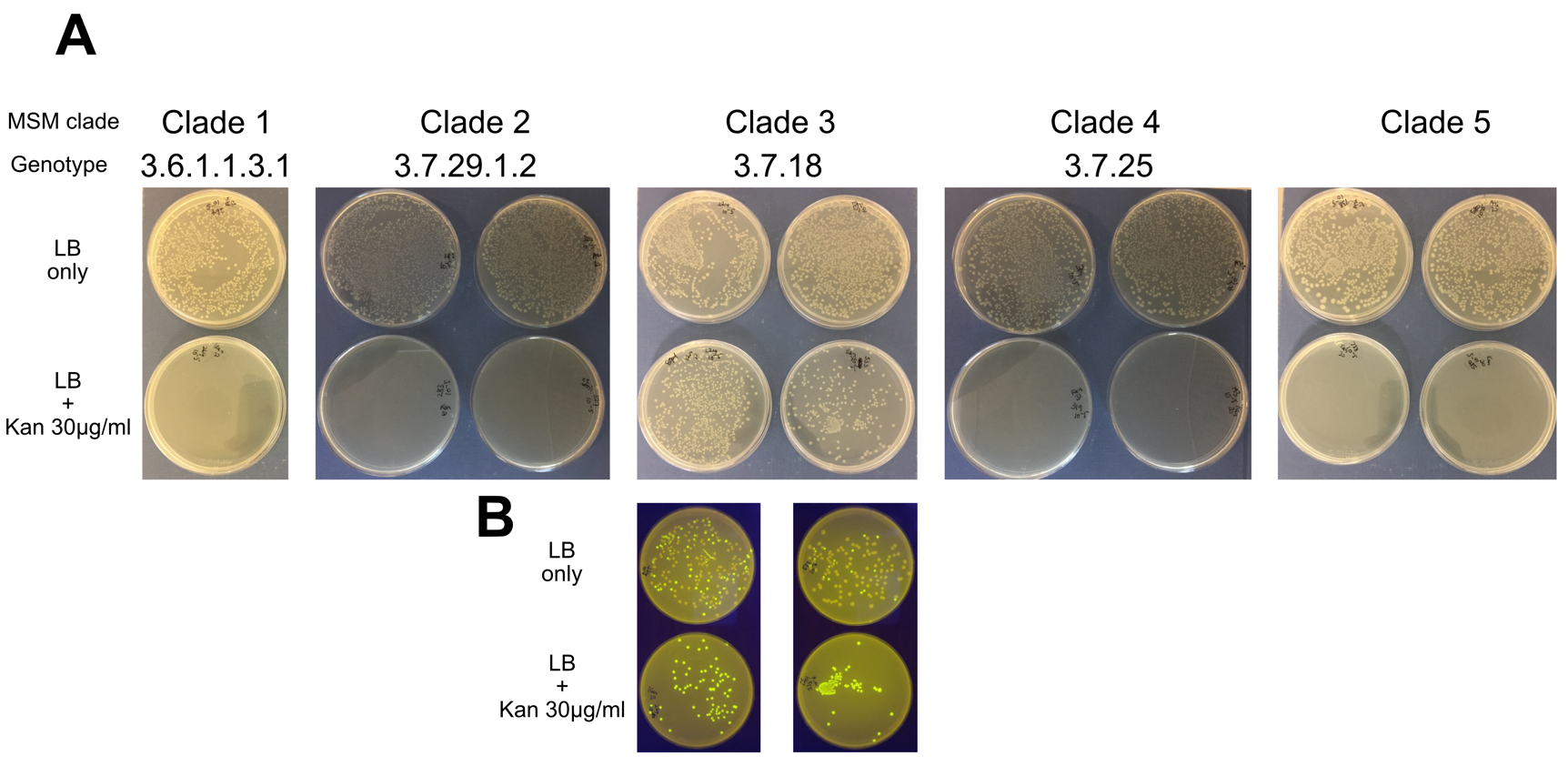


**Supplementary Figure 1. *E. coli* killing by representatives of *S. sonnei* MSM-associated clades.**

(A) Colony Forming Units on both non-selective (LB – top row) and selective (Kanamycin 30µg/ml – bottom row) for *E. coli* after the competition assay of individual representative isolates belonging to the different MSM associated clades showing the appearance of *E. coli* colonies when competed with only clade 3. (B) confirmation of the presence of *E. coli* colonies via the chromosomally encoded constitutively expressed GFP in *E. coli* MG1655 used in this study.


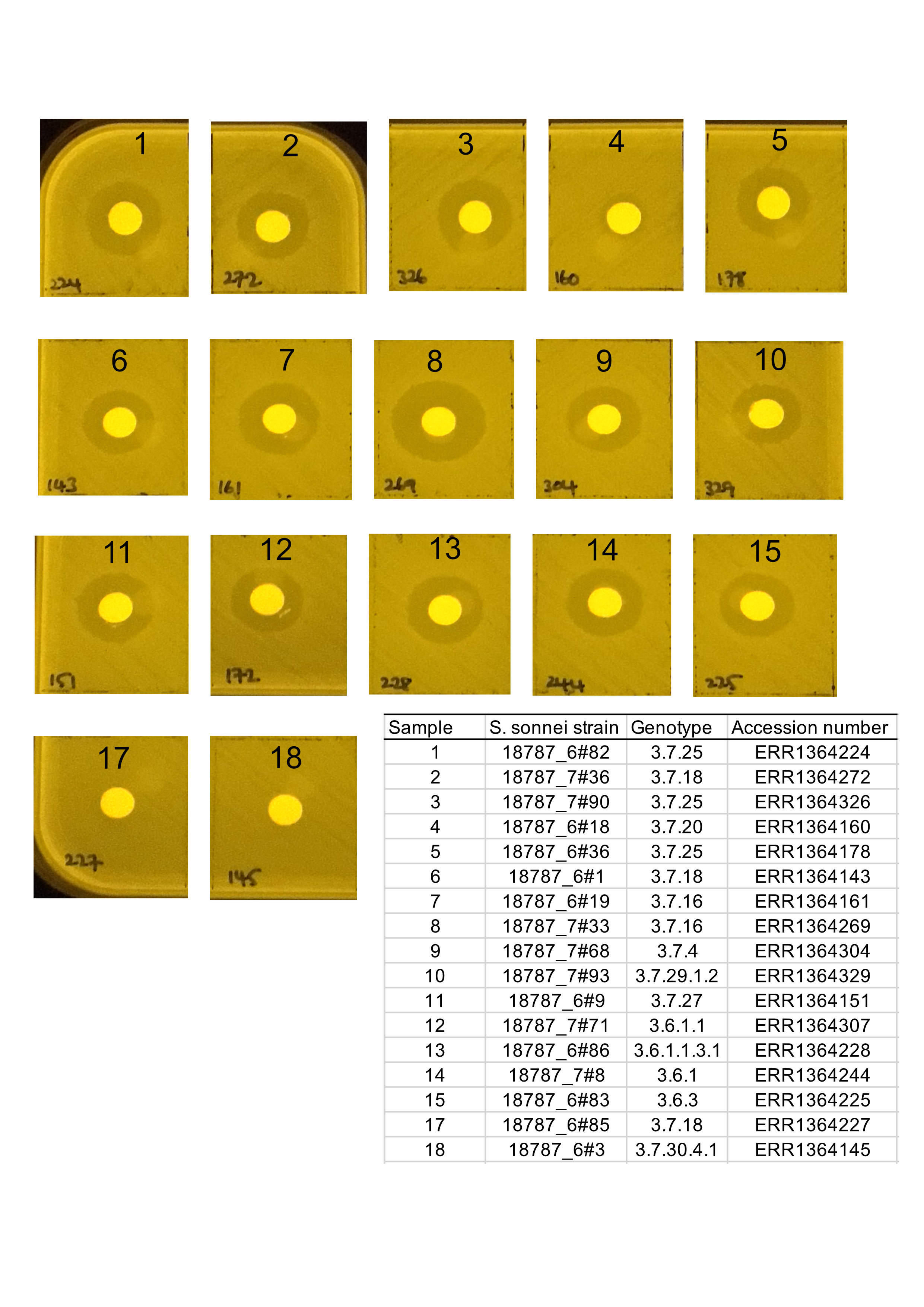


**Supplementary Figure 2. *E. coli* killing by the cell-free supernatants of representative *S. sonnei* isolates.**

*E. coli* killing as indicated by the zones of clearance of the *E. coli* lawn around the filter papers containing cell-free supernatant from selected *S. sonnei* isolates demonstrating *E. coli* killing via colicins present in the supernatants.

**Supplementary Figure 3. *S. sonnei* killing of four *E. coli* isolates including MG1655, and three clinical isolates.**

The tree is a rooted phylogeny of 163 clinical *S. sonnei* isolates whose genotype is shown in the adjacent colour strip according to the inlaid key. Sequential bar charts show the percentage of *E. coli* left following competition of *S. sonnei* against MG1655, *E. coli* LAE31441672004 (ST10), *E. coli* LAES2471692004 (ST6199), and *E. coli* LAE3140732004 from left to right. A cut off of 20% to introduce a proportional representation is indicated overlying each bar chat as a red line with the above numbers indicating the percentage of killing (left arrow) and non-killing (right arrow) observed for each *E. coli* isolate.


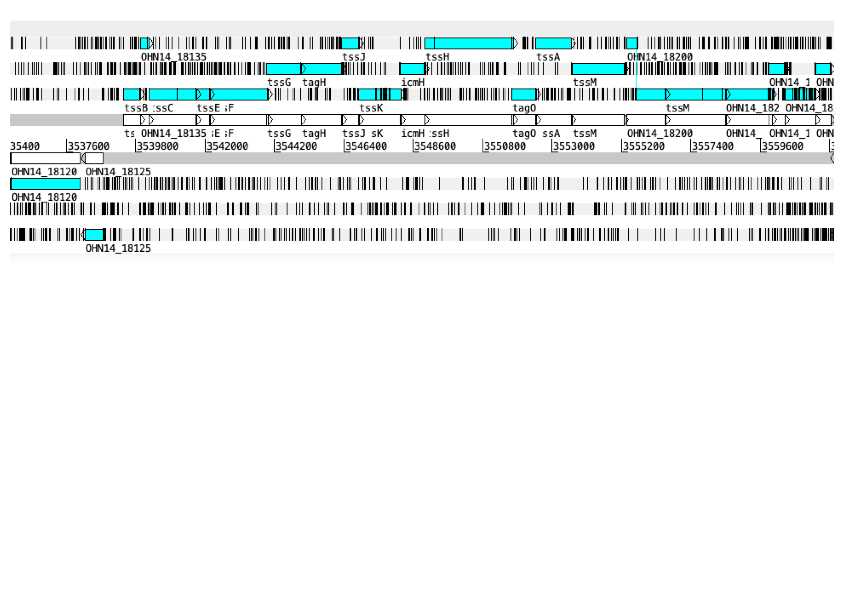


**Supplementary Figure 4. Organisation and the disruption of the T6SS gene cluster in isolate CIP 106347.**

Genetic organisation of the T6SS operon of sequenced CIP 106374 isolate (GenBank accession CP109775.1) showing the components of T6SS and the interruptions of genes involved. The top three tracks (+1, +2, +3) and the bottom three tracks (-1, -2, -3) indicate the reading frames with predicted coding sequences shown in turquoise block overlays (using automated NCBI Pathogens annotation pipeline). Vertical black lines in each track indicate stop codons. Notably, numerous predicted coding sequences contain stop codons and predicted CDS OHN14_18200 represents an insertion sequence interrupting *tssM*

**Supplementary tables provided as separate files**

**Supplementary Table 1.** **Isolate data used in this study.** Available metadata, sequence data accession numbers, and the presence/absence of key colicin clusters for *S. sonnei* strains used for the laboratory experiments and the reference isolate CIP 106347 along with correlation of the two BPER methods used to measure *E. coli* killing by *S. sonnei*.

**Supplementary Table 2.** Genes of interest identified for *E. coli* killing activity from GWAS analysis using PySeer and Scoary approaches based on the kmers and COGs (in default output format to be interpreted alongside program documentation).

**Supplementary Table 3.** Correlation of predicted and proteomic colicins among representative isolates.

**Supplementary Table 4**

Complete results of the mass spectrometry analysis of the *S. sonnei* representative isolates for the presence of colicins.

**Supplementary Table 5**

Complete list of identified colicins for all *S. sonnei* isolates used in the BPER experiments presented as the default output format of ARIBA.

**Supplementary Table 6**

Complete list of identified colicin and colicin related genes for each of the *E. coli* isolates used in the BPER assay presented in the default output format from ABRicate with the two leftmost columns indicating the strain of *E. coli* and the SRR accession number corresponding to the WGS data deposited in ENA.

**References**

1. Wick RR, Judd LM, Gorrie CL, Holt KE. Unicycler: Resolving bacterial genome assemblies from short and long sequencing reads. PLoS Comput Biol. 2017;13(6):e1005595.

2. Walker BJ, Abeel T, Shea T, Priest M, Abouelliel A, Sakthikumar S, et al. Pilon: an integrated tool for comprehensive microbial variant detection and genome assembly improvement. PLoS One. 2014;9(11):e112963.

3. Li H, Durbin R. Fast and accurate short read alignment with Burrows-Wheeler transform. Bioinformatics. 2009;25(14):1754-60.

4. Li H, Handsaker B, Wysoker A, Fennell T, Ruan J, Homer N, et al. The Sequence Alignment/Map format and SAMtools. Bioinformatics. 2009;25(16):2078-9.

5. Danecek P, McCarthy SA. BCFtools/csq: haplotype-aware variant consequences. Bioinformatics. 2017;33(13):2037-9.

6. Page AJ, Taylor B, Delaney AJ, Soares J, Seemann T, Keane JA, et al. SNP-sites: rapid efficient extraction of SNPs from multi-FASTA alignments. Microb Genom. 2016;2(4):e000056.

7. Croucher NJ, Page AJ, Connor TR, Delaney AJ, Keane JA, Bentley SD, et al. Rapid phylogenetic analysis of large samples of recombinant bacterial whole genome sequences using Gubbins. Nucleic Acids Research. 2014;43(3):e15-e.

8. Kozlov AM, Darriba D, Flouri T, Morel B, Stamatakis A. RAxML-NG: a fast, scalable and user-friendly tool for maximum likelihood phylogenetic inference. Bioinformatics. 2019;35(21):4453-5.

9. Letunic I, Bork P. Interactive Tree Of Life (iTOL) v4: recent updates and new developments. Nucleic Acids Research. 2019;47(W1):W256-W9.

10. Hunt M, Bradley P, Lapierre SG, Heys S, Thomsit M, Hall MB, et al. Antibiotic resistance prediction for Mycobacterium tuberculosis from genome sequence data with Mykrobe. Wellcome Open Res. 2019;4:191.

11. Hawkey J, Paranagama K, Baker KS, Bengtsson RJ, Weill F-X, Thomson NR, et al. Global population structure and genotyping framework for genomic surveillance of the major dysentery pathogen, *Shigella sonnei*. Nature Communications. 2021;12(1):2684.

12. Roche B, Garcia-Rivera MA, Normant V, Kuhn L, Hammann P, Bronstrup M, et al. A role for PchHI as the ABC transporter in iron acquisition by the siderophore pyochelin in *Pseudomonas aeruginosa*. Environ Microbiol. 2022;24(2):866-77.

13. Bouyssie D, Hesse AM, Mouton-Barbosa E, Rompais M, Macron C, Carapito C, et al. Proline: an efficient and user-friendly software suite for large-scale proteomics. Bioinformatics. 2020;36(10):3148-55.
